# Evaluating the acceptability of a co-produced and co-delivered mental health public engagement festival: Mental Health Matters, Jakarta, Indonesia

**DOI:** 10.1186/s40900-019-0161-3

**Published:** 2019-09-06

**Authors:** Helen Brooks, Irmansyah Irmansyah, Herni Susanti, Bagus Utomo, Benny Prawira, Livia Iskandar, Erminia Colucci, Budi-Anna Keliat, Karen James, Penny Bee, Vicky Bell, Karina Lovell

**Affiliations:** 1 Department of Health Services Research, Institute of Population Health Sciences, University of Liverpool, Liverpool, UK; 2 National Institute of Health Research and Development, Jakarta, Indonesia; 3 Marzoeki Mahdi Hospital, Bogor, Indonesia; 4 Faculty of Nursing, Universitas Indonesia, Depok, Indonesia; 5 KPSI, Jakarta, Indonesia; 6 Atma Jaya Catholic University of Indonesia, Jakarta, Indonesia; 7 Indonesian Agency for Witness and Victims Protection, Jakarta, Indonesia; 8 Pulih@thePeak- Women, Youth and Family Empowerment Center, Jakarta, Indonesia; 9 Department of Psychology, Middlesex University, London, UK; 10 Centre for Health and Social Care Research, Faculty of Health, Social Care and Education, Kingston and St Georges, London, UK; 11 Division of Nursing, Midwifery and Social Work, School of Health Sciences, Faculty of Biology, Medicine and Health, University of Manchester, Manchester Academic Health Science Centre, Manchester, UK; 12 Greater Manchester Mental Health NHS Foundation Trust, Manchester, UK

**Keywords:** Mental health, Public engagement, Research prioritisation, Patient and public involvement, Festival, Co-production

## Abstract

**Background:**

Public engagement events are an important early strategy in developing a meaningful research agenda, which is more impactful and beneficial to the population. Evidence indicates the potential of such activities to promote mental health literacy. However, this has not yet been explored in Indonesia.

**Aim:**

This paper describes a mental health public engagement festival carried out in Indonesia in November 2018 and uses evaluation data to consider the acceptability and use of such activities in Indonesia in the future.

**Method:**

Evaluation data was collected from 324 of the 737 people who attended a six-day mental health festival comprising 18 events including public lectures, film screenings, arts activities, exercise classes and panel discussions. Attendees were asked to evaluate the festival in terms of its quality, benefits and areas for improvement. Descriptive statistics were used to analyse the evaluation data. 87 service users, carers, academics and professionals also engaged in a research prioritisation exercise to collaboratively determine mental health research priorities for Indonesia.

**Results:**

Participants evaluated the festival extremely positively with a significant majority (92%) rating the quality of the festival as good or excellent. Attendees reported an increase in their understanding of mental health issues and identified intended behaviour change including an increased propensity for future engagement with mental health research. Key strengths of the festival included the central role of patients, carers and the local community in the design and delivery of the festival which promoted emotional engagement and development of shared understanding and the use of international experts which in attendees’ opinion further enhanced the credibility of festival activities.

**Conclusion:**

This manuscript indicates that a co-produced mental health public engagement festival is a potentially acceptable way to increase awareness of mental health in Indonesian populations. Future festivals should be larger in scope and target men, older people and the general public to maximise benefit and incorporate rigorous evaluation of effectiveness.

**Electronic supplementary material:**

The online version of this article (10.1186/s40900-019-0161-3) contains supplementary material, which is available to authorized users.

## Plain English summary

This commentary describes a six day mental health festival which was held in Jakarta, Indonesia in November 2018. The festival was organised and delivered by patients, family members, clinicians and academics with the aim of improving knowledge of mental health amongst attendees, agreeing future research priorities and encouraging involvement in mental health research in Indonesia. Festival activities included film screenings, public lectures, exercise classes, arts activities and question and answer sessions with people with personal experience of mental health problems. The festival included 18 different events, which were attended by 737 people. Eighty-seven service users, carers, academics and health professionals also engaged in a research prioritisation exercise to collaboratively determine mental health research priorities for Indonesia. We looked at anonymous evaluation data collected by the organising committee to see how useful people thought the festival had been. 92% thought the festival was good or excellent and people who attended the festival felt it had increased their understanding of mental health issues and most people felt they were more likely to get involved in mental health research in the future. Key strengths of the festival included the central role of patients, family members and the local community in the design and delivery of the festival. Suggestions for improvements for future festivals included having bigger festivals so more people could attend, trying to encourage more men and older people to attend to maximise benefit and to evaluate the festival in more detailed way (e.g. looking specifically at whether people’s behaviour changed after the festival). Working closely with patient and public representatives we also developed a film documenting festival activities which can be found here: https://stream.liv.ac.uk/cp3uchyb.

## Background

Mental health problems are a prominent cause of burden, accounting for 13% of the global burden of disease [1]. The impact of such conditions include reduced life expectancy and quality of life, increased social isolation and poorer physical health [2]. This burden also extends to those who care for people with mental health difficulties and to the wider society [3]. The United Nations' Sustainable Development Goals have advocated for the reduction of mortality by 1/3 by 2030 through the prevention and promotion of mental health and wellbeing and suicide prevention [4]. However, treatment gaps in most Low-Middle Income countries (LMIC) exceed 70% [5] which has led to calls to consider alternative forms of service provision and innovative ways to promote good mental health.

Indonesia, a nation of 268 million people in Southeast Asia, is classified as a LMIC according to World Bank Criteria. In line with other LMICs, mental health is now a priority in Indonesia and a national plan for minimum standards of mental health provision was developed in 2016 with mental health being one of the 12 healthy family indicators prioritised at a primary care level. However, significant treatment gaps persist due to insufficient funding and an inadequate level of sufficiently trained health workers [6]. Mental health literacy defined, as the ‘knowledge and beliefs about mental disorders which aid their recognition, management or prevention’ [7] is a critical mediator of health and functional outcomes [8]. In Indonesia, low levels of mental health literacy are thought to [9] contribute to delays in help seeking for mental illness [10, 11]. Low levels of awareness about and negative perceptions of mental health within communities further compound health service factors which have led to thousands of people being shackled or chained (‘pasung’) in the family home [12].

Having established research collaborations between Indonesia and UK in the form of research capacity building activities [13] and shared grant funding [9], a key early activity was to conduct a public engagement event with relevant stakeholders. Public engagement activities explore the meaning of research and aim to shape the research agenda. Public engagement has been defined as

“the myriad of ways in which the activity and benefits of higher education and research can be shared with the public. Engagement is by definition a two-way process, involving interaction and listening, with the goal of generating mutual benefit” [14].

Public engagement activities have been shown to promote mental health literacy and reduce the negative perceptions associated with mental illness amongst attendees reflecting a combination of education based activity and contact between the public and people with mental health conditions [15, 16]. However, there are limited published evaluations of mental health festivals generally and no published evaluations in Indonesia.

### Mental Health Matters 2019 public engagement festival

Our public engagement event utilised a six-day mental health festival at the Faculty of Nursing in the Universitas Indonesia in Jakarta with a range of activities including public lectures, film screenings (documentaries with the cast attending for question and answer sessions), arts activities, exercise classes and panel discussions in an attempt to maximise engagement with users of mental health services and their families, mental health organisations, academics and health professionals.

The festival was planned by our Indonesia/UK research collaboration organising committee consisting of mental health professionals, academics and representatives from four Mental Health Voluntary organisations in Indonesia, which included people with lived experience of mental illness and their families. The festival was aimed at adults and was free to attend. Tickets did not need to be obtained in advance. Given the nature of films and festival activities, attendees were told they could leave activities at any point and that they could talk to festival co-ordinators in another room if they wished. No attendees felt the need to do this during the festival. See Table 1 for more detail of festival activities.

**Table 1:** Festival activities and attendance

Session	Details	Attendance	Led by
12 November (am)			
Public lecture	4 lectures from Indonesian and UK mental health experts	122	Universitas Indonesia/Ministry of Health, Indonesia, University of Manchester
Introduction to Komunitas Peduli Skizofrenia Indonesia (KPSI)	Learn about the work of KPSI	122	KPSI, Indonesia
12 November (pm)			
Introduction to current research	Introduction to two ongoing mental health projects in Indonesia (IGNITE and IIMPETUS)	47	Ministry of Health, Indonesia
Memory Of My Face film screening and Q&A	Film screening of a patient story about the experience of and recovery from mental illness.	47	Health professionals and service user/family cast, Indonesia and film maker (University of Middlesex)
13th November (am)			
Interactive session on bullying	Hurt or Help: How to prevent bullying and suicide (sharing stories about bullying and suicide in young people)	32	Into the Light Indonesia, BTS Army Indonesia Amino, BTS Army Help Center
13th November (pm)	Invest in Youth Mental Health – Talk show series	30	Pulih at the Peak and Universitas Indonesia
14th November (am)	Research prioritisation event	87	Universitas Indonesia/University of Liverpool/University of Manchester
14th November (pm)			
Visual methods	Mental health training for journalists	47	University of Middlesex
Learn about our work	Learn about the work of Pulih at the Peak	47	Pulih at the Peak, Indonesia
	‘Breaking the Chains’ film screening and Q&A with cast	47	Service users, carers, community members (Indonesia) and film maker (University of Middlesex)
15th November (am)			
Exercise class	Sweat Out Your Stress (physical activity and mental health)	51	Into the Light Indonesia, Fit-BID and Manhunt Indonesia, Indonesia.
15th November (pm)	Poetry workshop	32	Pulih at the Peak, Indonesia
16th November (am)	Talk show event with family members of people who died by suicide	108	Mental Health Association, Indonesia
16th November (pm)	Learn about the work of Into the Light	64	Into the Light, Indonesia
	Film screening of patient story of experience of and recovery from pasung and Q&A session	64	Service user cast (Indonesia) and film maker (University of Middlesex)
17th November (am)	Film Screening Lalui Luka: A daughter’s journey and discussion with panel of service users, carers and professionals	117	Into The Light Indonesia, Faculty of Psychology at the University of Gadjah Mada, SHINee World Indonesia, and suicide loss survivors.
17th November (pm)	Digital exhibition of service user art	117	Faculty of Nursing, Universitas Indonesia - Ikatan Perawatan Kesehatan Jiwa Indonesia (IPKJI)
	Q&A session with service users and professionals about the experience of mental health	117	

Our Patient and Public Involvement partners included KPSI, a user-led charity, which runs peer support groups, education and anti-stigma programs with local health services and in the local community. Into the Light Indonesia is a youth based community which focuses on evidence-based suicide prevention and mental health promotion amongst young people and other high risk groups We also partnered with Yayasan Pulih (The Pulih Foundation), The Mental Health Association (Perhimpunan Jiwa Sehat), Indonesian Association of Psychosocial Rehabilitation and Indonesian Mental Health Nurses Association Jakarta (IPKJI) in Jakarta. All partners contributed to the organisation of the festival and delivered at least one event at the festival.

A short filming detailing festival activities can be found here: https://stream.liv.ac.uk/cp3uchyb along with an animation produced with the festival organising committee to further promote engagement in future mental health research in English: https://www.youtube.com/watch?v=BmzARp4n-G4 and Bahasa Indonesian: https://www.youtube.com/watch?v=Fi0-nvG%2D%2DCM

Aims of the festival:

1. To improve knowledge of mental health amongst attendees through a co-designed and co-delivered mental health festival
2. To strengthen relationships between community organisations, health services and higher education institutes and explore the potential for future festivals
3. To promote future engagement in mental health research
4. To identify future mental health research priorities

Aims of the evaluation:

1. To explore the impact of the festival on knowledge/understanding of mental health and future behavioural intentions
2. To develop understanding on the acceptability of undertaking mental health festivals in Indonesia to raise awareness of mental health
3. To identify any behavioural intentions related to future engagement in mental health research

## Methods

Evaluation forms were designed in collaboration with the organising committee and distributed at every event during the festival to all attendees (see Additional file 1 for the evaluation form). Evaluation forms comprised both structured and unstructured questions and were completed anonymously. Participants left questionnaires in pre-arranged boxes on campus prior to leaving the festival.

### Structured question

The questionnaire included demographic information such as gender and age as well as how the attendee found out about the festival. It also captured data on the perceived quality of the events and the impact of the festival on knowledge and future behavioural intentions related to mental health research. Questions were informed by a review of the literature including previous evaluations of mental health public engagement activity [15]. Survey responses were entered into SPSS and analysed descriptively using frequency of responses, mean and range.

### Unstructured questions

Unstructured questions explored why participants attended the festival, what they liked and did not like about the festival and what they would like to see improved in future festivals:

- Why did you attend?
- What did you like about the event?
- What did you not like about the event?
- Do you have any suggestions for improvement?

Unstructured questions were analysed using principles of content analysis, an analytical technique that enables large amounts of textual responses to be managed and organised [17]. This method is appropriate for use with short and varied responses akin to the responses to the unstructured questions within the evaluation form [18]. The responses were first read and reread in their entirety before the researcher allocated codes to each response. Codes were organised into overarching categories with duplicate codes removed and similar codes combined. Overarching categories were then described narratively and presented with supporting responses taken from the evaluation forms. The presentation of unstructured survey response categories was shared with the wider research team to ensure they reflected the data on which they were based.

#### Research prioritisation exercise

On the third day of the festival, 87 service users, carers, academics and professionals engaged in a research prioritisation exercise to collaboratively identify future mental health research priorities for Indonesia. The approach was informed by the Guidance for Priority Setting Partnerships [19] and the Checklist for Health Research Priority Setting: Nine Common Themes of Good Practice [20].

Preparatory work was undertaken by the organising committee, made up of service users, carers, academics and health professions, who were responsible for the priority setting exercise [19]. They decided on the resources available, who should be invited and the approach to be undertaken. The organising committee invited relevant stakeholders to ensure balanced representation from service users, carers, academics and health professionals utilising existing networks.

The research prioritisation exercise was introduced by members of the organising committee along with the methods to be undertaken, the expected outcomes and who would take forward identified priorities to ensure transparency of process [19]. Attendees split themselves into 10 groups which each included a mix of different stakeholders.

##### Stage 1

Groups were given 90 min to discuss their thoughts and generate ideas for research priorities related to future mental health in Indonesia. There was no limit on the scope or number of research priorities that groups could identify. Attendees were asked to write identified priorities on flip chart paper for audit trail purposes [19]. Members or the organising committee were available during the process should attendees have questions or queries relating to the process.

##### Stage 2

To reduce the list of identified priorities to a shorter list to be voted on by all attendees, each group then had 30 min to discuss identified priorities and select one of their generated ideas to take forward. Consensus was required amongst the whole group in order for a priority to be taken forward to stage 3.

##### Stage 3

The 11 identified research priorities (one additional priority was identified as the groups fed back) were compiled onto flip chart paper and presented to all attendees. The group agreed that identified priorities were different enough from each other to stand-alone for stage 4 voting.

##### Stage 4

Each group were allocated 2 votes, which they could use on any of the identified research priorities to determine three top priorities within the set of 11. Groups had 30 min to discuss and reach consensus on this. A nominated member from each group was then given a marker pen to indicate the group’s votes by placing ticks next to the two chosen priorities. A member of the organising committee oversaw this process to ensure each group only cast two votes.

##### Stage 5

Scores were calculated for each priority and the three research areas considered to be of greatest priority by the group as a whole were announced. The organising committee reiterated their responsibility for taking these forward by publishing and developing research proposals accordingly before the exercise closed.

## Results

There were a total of 18 events at the six-day festival, attracting 737 attendees. 324 of the 737 (43.9%) attendees completed and returned evaluation forms. The events with the highest attendance were the public lectures, the panel discussions/talk shows/Q&A sessions with service users and carers and film screenings.

### Structured questions

The majority of attendees who completed the evaluation form were female (89% vs. 11%) and the mean age of attendees was 22.5 years, ranging from 17 to 51. Students (50%) and patients and public attendees (20%) made up the majority of the audience over the 6 days. Most participants found out about the event via social media or from people they knew. The main reasons for attending the festival included a general interest in the topic of mental health and a desire to increase their knowledge and experience related to mental health (Table 2).

**Table 2:** Demographic data of attendees

	% (*n*)
Gender	
Female	88.6% (*n* = 286)
Male	10.8% (*n* = 35)
Age	
Mean	22.5 years
Range	17–51 years
Role	
Student	50.3% (*n* = 163)
Patient or public attendee	20.4% (*n* = 66)
Professional	5.3% (*n* = 17)
Missing	24.1% (*n* = 78)
How attendees heard about the festival	
Social Media	28.4% (*n* = 92)
Colleagues	28.1% (*n* = 91)
Poster/flyer	5.6% (*n* = 18)
Other organisation	9.0% (*n* = 29)
Other	21.9% (*n* = 68)
Missing	8.0%(*n* = 26)
Reasons for attending	
Interest in or relevance of topic	46.9% (*n* = 152)
Wanted to increase knowledge and experience	32.1% (*n* = 104)
Invited	6.5% (*n* = 20)
No specific reason	4.0% (*n* = 13)
To share experience/represent community	3.4% (*n* = 11)
Opportunity to hear named speakers	3.1% (*n* = 10)
Missing	4.3% (*n* = 14)

Table 3 shows the overall evaluation for the festival. All questions demonstrated extremely positive evaluations of the festival. 92% (*n* = 299) rated the overall quality of the festival as good or excellent. In terms of knowledge, 80% reported an increase in their understanding of mental health problems. 72% reported intended behaviour change by stating they were more likely to get involved in health research after attending the festival.

**Table 3:** Festival evaluation

	% (*n*)
Overall quality rating of festival	
Excellent	6.5% (*n* = 21)
Good	85.8% (*n* = 278)
Average	4.9% (*n* = 16)
Poor	0.3% (*n* = 1)
Missing	2.5% (*n* = 8)
Total	324
Has it increased your understanding of mental health problems?	
Yes	79.9% (*n* = 259)
No	0.9% (*n* = 3)
Missing	19.1% (*n* = 62)
Total	324
Has it increased the likelihood that you would get involved in health research in the future?	
Yes	72.2% (*n* = 234)
No	2.5% (*n* = 8)
Unsure	21.6% (*n* = 70)
Missing	3.7% (*n* = 12)
Total	324
I felt moved or inspired	
Strongly agree	18.5% (*n* = 60)
Agree	70.7% (*n* = 229)
Neither agree nor disagree	6.5% (*n* = 21)
Disagree	0.9% (*n* = 3)
Missing	3.4% (*n* = 11)
Total	324
I felt engaged in the experience	
Strongly agree	14.2% (*n* = 46)
Agree	63.6% (*n* = 206)
Neither agree nor disagree	18.5% (*n* = 60)
Disagree	0.3% (*n* = 3)
Missing	3.4% (*n* = 11)
Total	324
I was exposed to new points of view or ways of thinking about things	
Strongly agree	20.7% (*n* = 67)
Agree	68.8% (*n* = 223)
Neither agree nor disagree	6.5% (*n* = 21)
Disagree	0.3% (*n* = 1)
Missing	3.4% (*n* = 11)
Total	324
It made me want to know more about what I was seeing	
Strongly agree	24.1% (*n* = 78)
Agree	63.6% (*n* = 206)
Neither agree nor disagree	7.7% (*n* = 25)
Disagree	0.6% (*n* = 2)
Missing	3.7% (*n* = 12)
Total	324
It felt relevant to our society and the times we live in	
Strongly agree	33.3% (*n* = 108)
Agree	60.8% (*n* = 197)
Neither agree nor disagree	2.2% (*n* = 7)
Disagree	0.3% (*n* = 1)
Missing	3.4% (*n* = 11)
Total	324

Identified benefits included emotional engagement in activities (89% of attendees felt moved or inspired during festival events and 78% of attendees felt engaged in festival activities) and festival material challenging existing ways of thinking (90% agreed they had been exposed to new view points and new ways of thinking). 94% felt the festival was directly relevant to their lives and the times in which they lived and 88% wanted to find out more about mental health related issues as a result of attending the festival.

### Unstructured questions

The topic of mental health was considered by attendees to be important in Indonesia and directly relevant to people’s everyday lives and the communities in which they lived. As such, attendees described welcoming the opportunity to attend an event dedicated to this topic.

*The issues [discussed] are real and affect real life communities.* ***ID44, Female, aged 22.***

*The speakers were very inspiring, the topic presented was very interesting and very useful for me, my family and the community* ***ID251, Female, aged 22***

Attendees reported that the festival enhanced their knowledge and understanding of mental health related issues. The combination of expert international speakers with local community representatives and people with lived experience of mental illness contributed to the credibility of the event and facilitated knowledge enhancement. The use of a well-known university campus was also positively received by participants. Participants valued having the opportunity to share experiences with others and learn new things during the interactive sessions.

*This event increased my understanding of mental health problems and brought people together who care about mental health* ***ID24, Female, aged 22.***

*[What I liked about the festival was that] film screenings were held in a well-known campus with people who have expertise in filmmaking.* ***ID279, Male, aged 34***

*This event is very good because it encourages you to think about mental health more.* ***ID121, Female, aged 20***

Of particular value to attendees was the opportunity to engage with and hear from community and voluntary organisations and people with lived experience across a range of different festival activities including lectures, film screenings, exercise classes and poetry workshops.

*The topic is very touching. It shows that being diagnosed with mental illness is not the end of things.* ***ID203, Female, aged 18***

*Hearing from someone who has experienced mental illness (talking & answering), inspiring!* ***ID274, Female, aged 22***

Arts-based activities and in particular the film screenings were viewed positively and described as both inspirational and motivational. The fact that attendees were able to interact and engage with cast members after the film was especially well received.

*This event [film screening] featured someone who had experienced [mental health issues] directly, but now has become an inspiring person.* ***ID200, Female, aged 18***

*Nice films that were inspiring!* ***ID298, Male, aged 19***

The main suggestions for improvement focussed on the size of the event and the venue the festival was hosted in. Given that this was the first event of its kind in Jakarta, the scope was necessarily small. The room allocated to the festival was often not big enough to accommodate all the people who wished to attend and was occasionally cramped for those who did manage to make it into the room. The volume of attendees contributed to noise levels which could, at times, make hearing speakers difficult. There were some technical issues with speakers that reduced the impact of some of the films.

Participants felt that future festivals should be held regularly, run for longer, and have a greater capacity for attendees to maximise potential benefit. Wider publicity and a larger more accessible venue (e.g. central Jakarta) were considered necessary to encourage attendance. With more preparation time, the festival could have more participants (partners) and more events or performances

### Research prioritisation exercise

Identified research priorities can be found below. The first three were ranked as the top three priorities in the order presented and the rest are presented in no particular order.

1. Improve employment opportunities for people with mental health problems
2. Health promotion through information provision and campaigning to reduce the stigma related to mental health problems
3. Increase the mental health literacy of children and young adolescents

- Easy and simple procedures to access care (including health insurance)
- Understand the best way to educate people about mental health (most efficacious and cost-effective)
- Cadre-led community training to reduce pasung (the physical constraint and confinement of people with mental health problems)
- Increase the number of mental health professionals to increase access to care
- Equal distribution of medication and professionals
- Improve the mental health of students (particularly students from different areas)
- Social media and other forms of public health approaches to increase awareness of mental health amongst general population
- Social media and other forms of public health approaches to increase awareness of mental health amongst children and young people

## Discussion

Public engagement festivals incorporating arts and education based activities are increasingly being used within Western countries to improve mental health literacy [21]. Despite some evidence of effectiveness of these approaches, published evaluations are distinctly lacking [22]. This manuscript reports on the acceptability and feasibility of utilising such an approach in an Indonesian context. The Mental Health Matters festival, held in Indonesia in November 2018, was well attended with the number of attendees far exceeding our initial expectations. Evaluation data were overwhelmingly positive with identified impacts on knowledge and future behavioural intention related to engagement in mental health research. Participants reported being emotionally engaged with the festival and reported the construction of shared knowledge and understanding. Attendees felt strongly that the festival was of direct relevance to their lives and the communities in which they lived. Such findings are an encouraging indication of the acceptability of this type of public engagement activity in Indonesia.

Attendees identified the film screenings and panel discussions as of particular value highlighting the benefits of being able to engage with the cast members. Such findings support previous evaluations of film festivals which have demonstrated the capacity of films documenting real life experiences to promote critical reflection and shape cultural understanding of mental health when combined with panel discussions [23] and the impact of film making on the protagonists themselves [24]. Such interactive sessions with patients, carers and professionals were considered to be a key strength of the Mental Health Matters Festival.

The majority of participants who attended the festival found out about it via social media with very small numbers getting it know about it through more traditional methods (e.g. paper flyers). This highlights the utility of such approaches for engaging with Indonesian communities and reflects the high social media use in Indonesia more generally. Such approaches are likely to be fundamental to the success of future festival related activities and should play a central role in engagement strategies.

The research prioritisation exercise, which successfully engaged 87 service users, carers, academics and health professionals, collaboratively identified 11 mental health priorities for Indonesia whilst also reaching consensus on the three research areas considered to be of most importance; improving employment opportunities for people with mental health problems, health promotion through information provision and campaigning to reduce the stigma related to mental health problems and increased mental health literacy of children and young adolescents. The prioritisation exercise appeared to work particularly well in the context of the wider mental health festival. This may have been a result of the benefits people reported of attending the festival more generally (e.g. increased understanding of mental health issues and the propensity for increased engagement in mental health research) which is likely to be relevant to people tasked with planning similar events. Future prioritisation exercises could also consider promoting engagement from a wider audience through the use of Twitter and online surveys [19].

Demographic data collected during the evaluation process demonstrates that the majority of attendees were women and younger people. Previous research also indicates that people from higher socio-economic status are usually overrepresented at arts based activities generally [15]. These groups are considered to have greater mental health literacy and as such more positive attitudes towards those with mental illnesses which may limit the transferability of results [25]. Future festivals should try to target recruitment towards older people, men and members of the general public to maximise potential benefit. Attendees also felt that a larger venue in a more accessible venue (e.g. central Jakarta) would facilitate such developments.

Low mental health literacy has been identified as an important barrier to accessing mental health services and recovery from mental illness in Indonesia [12]. For example, it is a commonly held perception that mental illnesses result from supernatural influences which has a resultant impact on help seeking, treatment and decisions by family members to physically restrain people with mental illnesses in sheds or cages in the family home (pasung) [26]. Recent systematic reviews indicate that public engagement activities which provide education and increase contact with people with mental illness are a potentially effective way to reduce the discrimination associated with mental illness [22] This evaluation provides the first evidence that such approaches are acceptable to Indonesian populations. Most suggestions for improvement related to increasing the size and scope of the festival and holding such events more regularly.

Of particular value were the invited international experts and the central role of service users, carers and members of the local community in the design and delivery of the festival. The arts-based activities were considered especially inspiring and challenged perceptions about the trajectory of mental health conditions and the capacity of patients and carers in line with other studies [23]. However, not all previous mental health festivals in other parts of the world have shown universally positive effects and one such activity was shown to increase negative perceptions about those with mental health conditions [15]. This highlights the importance of adequate evaluation and the need to ensure activities are carefully considered and planned collaboratively with patients, their carers and the wider community.

Most participants attended the festival due to an interest in the topic and a desire to learn more about mental health in Indonesia and internationally. Evaluation outcomes were largely congruent with these expectations with a large majority of attendees saying that their knowledge had increased following attendance. Whilst it is not possible to identify actual behavioural change, there are promising signs within the evaluation data that attendees were motivated to change their behaviour following attending the festival. This included a greater propensity to engage in mental health research, a desire to find out more about mental health and the organisations that were involved in the festival. These outcomes link closely with the aims of the festival but larger scale evaluations of future festivals would be required to fully explore whether intentions manifest in actual behaviour change. Due to the success of this initial festival, the authors are planning to hold further events on a larger scale in the future and are currently looking for funding to support this. Results of this evaluation will inform the design and implementation of subsequent activity. Future festivals will include more in-depth evaluation to examine the impact of festival activities on behaviour change.

## Conclusion

Co-produced and co-delivered mental health public engagement festivals which incorporate education and arts-based activities are a potentially acceptable way to increase awareness of mental health in Indonesian populations. Such activities can contribute to increased understanding of mental health related issues and are a feasible option for health services to promote public mental health.

## Additional file

Additional file 1: Festival Evaluation Form. (DOCX 14 kb)

## Data Availability

The datasets used and/or analysed during the current study are available from the corresponding author on reasonable request.

## Abbreviations

IPKJI: Ikatan Perawatan Kesehatan Jiwa Indonesia
KPSI: Komunitas Peduli Skizofrenia Indonesia
LMIC: Low-Middle Income countries
Q&A: Questions and answers
SPSS: Statistical package for the social sciences
UK: United Kingdom
