# Supplementary material for: Evaluating the acceptability of a co-produced and co-delivered mental health public engagement festival: Mental Health Matters, Jakarta, Indonesia
Source: Res Involv Engagem. 2019 Sep 6;5:25. doi: 10.1186/s40900-019-0161-3 (PMC6728994; doi:10.1186/s40900-019-0161-3)
Supplement: Supplementary file 1 — Festival Evaluation Form. (DOCX 14 kb) [file 40900_2019_161_MOESM1_ESM.docx]

**Additional file 1:**

**MENTAL HEALTH MATTERS FESTIVAL – NOVEMBER 2018 – EVALUATION FORM**

We would like to ask you a few questions about your experience of this event. Your responses are confidential and you will not be identified in the survey results. Please be open and honest with your feedback as this will help us with our future planning.

1. Gender:

🞏 Male 🞏 Female 🞏 Other: _______________

2. How old are you? ___________

3. Organization _____________

4. Role______________

4. How did you find out about the festival?

🞏 Social Media 🞏 Poster Flyer 🞏 Colleagues

🞏 Organisation: ____________________________

🞏 Other: ______________________________­­

5. Why did you attend?

_______________________________________________________________________________________________________________________________________________________________________________________________________________________________________________________________________________________

6. Which event(s) did you attend?

____________________________________________________________________________________________________________________________________________________________________________________________________________________________________________________________________________________

7. How would you rate the quality of the festival:

🞏 Excellent 🞏 Good 🞏 Average 🞏 Poor 🞏Very poor

8. Has attending increased your understanding of mental health problems?

9. Has it increased the likelihood that you would get involved in health research in the future?

🞏 Yes 🞏 No 🞏 Unsure

10. How would you rate the festival overall?

🞏 Excellent 🞏 Good 🞏 Average 🞏 Poor 🞏Very poor

Please indicate the extent to which you agree or disagree with the following statements based on your experience of the festival.

|  | Strongly agree | Agree | Neither agree nor disagree | Disagree | Strongly disagree |
| --- | --- | --- | --- | --- | --- |
| I felt moved or inspired |  |  |  |  |  |
| I felt engaged in the experience |  |  |  |  |  |
| I was exposed to new points of view or ways of thinking about things |  |  |  |  |  |
| It made me want to know more about what I was seeing |  |  |  |  |  |
| It felt relevant to our society and the times we live in |  |  |  |  |  |

11. What did you like about the event?

____________________________________________________________________________________________________________________________________________________________________________________________________________________________________________________________________________________________________________________________________________________________________________________

12. What did you not like about the event?

____________________________________________________________________________________________________________________________________________________________________________________________________________________________________________________________________________________________________________________________________________________________________________________

13. Any suggestions for improvement?

____________________________________________________________________________________________________________________________________________________________________________________________________________________________________________________________________________________________________________________________________________________________________________________
